# A Novel Approach to Determine the Prevalence of Type of Soft Palate Using Digital Intraoral Impression

**DOI:** 10.1155/2017/3268064

**Published:** 2017-08-29

**Authors:** Saurabh Chaturvedi, Mohamed Khaled Addas, Abdullah Saad Ali Al Humaidi, Abdulrazaq Mohammed Al Qahtani, Mubarak Daghash Al Qahtani

**Affiliations:** ^1^Department of Prosthodontics, College of Dentistry, King Khalid University, Abha, Saudi Arabia; ^2^College of Dentistry, King Khalid University, Abha, Saudi Arabia

## Abstract

**Aim:**

To determine the prevalence of type of soft palate in targeted population.

**Materials and Methods:**

Using computer technology in dentistry, intraoral digital scanner, and 3D analysis software tool, study was conducted. 100 patients selected from the outpatient clinics were divided into two groups based on the ages of 20–40 years and 41–60 years with equal ratio of males and females. Each selected patient's maxillary arch was scanned with intraoral scanner; images so obtained were sectioned in anteroposterior cross section and with the 3D analysis software; the angulation between hard and soft palate was determined.

**Results:**

The prevalence of type II soft palate (angulation between hard and soft palate is between 10 and 45 degrees) was highest, 60% in group 1 and 44% in group 2. The difference between genders was statistically significant with *p* value <0.05 in both the groups, although females had higher angulation compared to the males in all classes of both groups.

**Conclusions:**

In targeted population of Aseer Province, Saudi Arabia, the prevalence of type II soft palate was more common, with higher soft palate angulation among females. The advanced age had no effect in the type of soft palate in the region.

## 1. Introduction

The soft palate is posterior fibromuscular part of the palate that is attached to the posterior edge of the hard palate. It participates in most oral functions, especially velopharyngeal closure which is related to the normal functions of sucking, swallowing, and pronunciation. Early references concerning the objective measurements of the soft palate have been contributed by investigators interested in speech function and upper airway structures [1–6].

The soft palate is a muscular fold suspended from the posterior border of the bony palate and extending downwards and backwards into the oropharynx. The five muscles of the soft palate play important roles in swallowing and breathing. The muscles are tensor veli palatini, palatoglossus, and levator veli palatini involved in swallowing, palatopharyngeus, involved in breathing and involved in swallowing, and musculus uvulae which moves the uvula. These muscles are innervated by the pharyngeal plexus via the vagus nerve, with the exception of the tensor veli palatini. The tensor veli palatini is innervated by cranial nerve 5 branch V3 (which is the mandibular division of the trigeminal cranial nerve) [1]. Usually, the soft palate and tongue are in tight apposition, closing the oropharyngeal isthmus; the soft palate can however rise and touch the posterior pharyngeal wall, closing the nasopharynx: thus the soft palate regulates the flow of air through nose and/or mouth. During oronasal breathing (as during exercise, speech, or smoking) the impedance of nasopharynx and oropharynx, respectively, is determined by the position of the soft palate [1, 3–5].

Although continuous efforts towards the dimensional analysis of the soft palate and its surrounding structures have been made, little attention has been paid towards the angulation between soft palate and hard palate. Along with the variety of soft palate morphology [2] and configuration, its angulation with hard palate is also important, as this angulation plays a very important role while establishing posterior palatal seal in complete denture construction [5, 6], during repair of soft palate cleft, while repositioning the muscular velum [3] (especially the levator veli palatini muscle) to allow for velopharyngeal function and for speech and swallowing.

Obstructive sleep apnea (OSA) is characterized by the recurrent occlusion of the upper airways resulting from the inspiratory collapse of the pharyngeal walls during sleep [7]. Etiological or predisposing factors for OSA are still debatable. There is an increased incidence of OSA in middle-aged adults. It was found that pharyngeal morphology is not immutably established during childhood and adolescence but changes throughout adult life. There is a tendency towards longer and thicker soft palate and narrower oropharynx during adulthood, which may explain the possible increased incidence of OSA and related disorders occurring later in life [8].

Velopharyngeal closure is obtained by normal apposition of the soft palate with the posterior and lateral pharyngeal walls, thus separating the oral cavity from the nasal cavity during deglutition and speech. When the velum and lateral and posterior pharyngeal walls fail to separate the two cavities, velopharyngeal incompetence (VPI) occurs. Nakamura et al. [9] reported that patients with persistent VPI had a shorter velar length and greater pharyngeal depth resulting in less than 100% length/depth ratio, which averaged more than 100% in velopharyngeal competent subjects. You et al. [10] considered that a harmony between velar length and pharyngeal depth may contribute to normal velopharyngeal functions. Wada et al. [11] found an adequate ratio (velar length to pharyngeal depth) of about 1.3 to 1.4 in normal subjects to maintain velopharyngeal closure during speech. However, in unilateral cleft lip and cleft palate patients, it was high at an early age but gradually decreased to about 1.1 which was significantly less than the noncleft control group at age of 17 years. The lesser value implied that the soft palate must move a greater distance to be in apposition with the posterior pharyngeal wall for attaining velopharyngeal closure. This may be the cause of reappearance of nasality at a later age in cleft palate repair patients who had a good speech earlier.

During the fabrication of prosthetic appliance for obstructive sleep apnea (OSA), palatopharyngeal incompetence, palatopharyngeal insufficiency, and palatopharyngeal inadequacy, how much lifting of the soft palate will be required and/or how much extension of the prosthesis will be needed to provide the closure in nasopharynx area will be determined by considering the angulation between soft and hard palate [2, 3, 7, 9–18]. During maxillofacial reconstructive surgeries, in cases of road traffic accidents, this angulation is beneficial. Cohen et al. [7] suggested that one of the several explanations for this surgically successful yet functionally compromised repair may be due to difference in morphology of soft palate and other associated structures in these patients than normal. Hence, presurgical evaluation of soft palate morphology aids in the success of surgery.

On the basis of angular relationship, soft palate is classified as type I, II, or III depending upon the angle formed by soft palate with hard palate, that is, type I > 10, type II = 10–45, and type III > 45 degrees. This angular relationship is called palatal throat form [12–14].

During intraoral examination, little attention is paid to determining the type of soft palate. Mostly while determining posterior palatal seal in complete denture fabrication, the soft palate angulation with hard palate is examined. This examination is done by visual method using imaginary lines around that area, which results in improper diagnosis and many more related effects are mistreated [14–16].

Methods for assessment of soft palate form include visual inspection, radiographic visualization, and cast assessment [19]. All these methods of assessment do not give exact readings for angulations and moreover observer bias is of concern. During visual inspection the observer will only have anteroposterior view; determination of angulation in this view mainly depends on experience of the observer. Also in order to get a perfect view sometimes patient is asked to open his mouth wide which may result in activation of soft palatal muscles causing difficult assessment of angulation. Radiographic evaluation itself comes with inherent radiographic error such as distortion of the image, blurring or loss of sharpness, positional errors, and overlapping of images, and above all the radiolucency of the soft tissue makes it difficult to determine the soft tissue margins. Cast assessment for determination of angulation is of very limited value because the moment the impression material contact the soft palate the activation of the muscles take place and angulation gets change.

The development of intraoral scanners has opened various channels in dentistry. A completely new era of impression making without any material has started. Although the first intraoral digitizers were commercially available 2 decades ago, it is only recently that the popularity of these systems has grown [20–26]. In the past 5 years, 5 new intraoral digitizers have been developed and successfully introduced with increased accuracy. But even after its use in dentistry for several years most information regarding their accuracy is limited to single elements commonly used in restorative dentistry, such as crowns, inlays, onlays, and bridges [24, 25].

The use of intraoral scanner in field of diagnosis and treatment planning is still unfolded. Moreover its use in determination of type of soft plate and its angulation with palate is not even in consideration. To our knowledge, no study till now has used the intraoral scanner to determine type of soft plate, even though it can assess the angulation of soft palate with hard palate using mathematical software with full accuracy without bias.

In the present study, the intraoral scanner for making digital impression was used, for the first time to determine the relation between the hard and soft palate. The aim of our study was to determine the prevalence of type of soft palate in the targeted population of Aseer Province of Saudi Arabia. The objectives were to determine the prevalence type of palatal throat form in young population, group 1 (20–40 years), and in aged population, group 2 (41–60 years). The determination of this prevalence will be helpful for the targeted population and surrounding areas of Saudi Arabia because, in Saudi Arabia, nearly 355,000 children are born each year (Ministry of Health, 2008, https://www.ncbi.nlm.nih.gov/pmc/articles/PMC3723074/). There is a high rate of consanguineous marriage, which could be indicative of a high prevalence of various congenital anomalies [27]. BaHammam et al. [28], in 2008, reported that one in 3 middle-aged Saudi males is at risk of OSA and high rates of road traffic accidents (RTAs) are responsible for many traumatic injuries in Saudi Arabia. Only in Aseer region about 140 maxillofacial trauma cases are reported per year, and the incidence of RTAs is similar to those in other developing countries [29, 30].

## 2. Materials and Method

This study was conducted in King Khalid University College of Dentistry (KKUCOD), Abha Province, Saudi Arabia.

### 2.1. Ethical Considerations

This study was conducted in compliance with the protocol; written ethical approval was taken from the ethical committee of the King Khalid University College of Dentistry. The subjects participating in the present study provided their informed consent. Participation was on a voluntary basis and there were no incentives. Data protection and concealment were guaranteed.

For this study, the samples were collected from the KKUCOD, outpatient department (OPD), in a period of three months. During this period, all patients reporting to the OPD were checked by a single examiner (who was blinded from the study). The examiner selected the samples only on the basis of inclusion and exclusion criteria from the pool of patients in OPD with the age range of 20–60 years. A total of 100 samples were collected. Two groups were made on the basis of age as groups 1 and 2 (Table 1).

Samples were collected with following inclusion and exclusion criteria.

### 2.2. Inclusion Criteria

The inclusion criteria followed were

1. cooperative patient;
2. patient with sufficient (interincisal opening 22–24 mm) mouth opening as limited mouth opening may hinder the placement of intraoral scanner and correct recording may be difficult;
3. nonobese patient (a/c body mass index: the body mass index (BMI) is a value derived from the mass (weight) and height of an individual; the BMI is defined as the body mass divided by the square of the body height and is universally expressed in units of kg/m2; a BMI from 18.5 up to 25 kg/m2 may indicate optimal weight; a BMI lower than 18.5 suggests the person is underweight; a number from 25 up to 30 may indicate the person is overweight; and a number from 30 upwards suggests the person is obese) as in obese patient the chances of OSA is more resulting in changed angulation between soft and hard palate;
4. patient with proper speech and pronunciation: any speech deformity may result in altered speech pattern and adaptation of oral musculature and change in tonicity of muscles of soft palate.
5. patient without any history of previous maxillofacial trauma.

### 2.3. Exclusion Criteria

The exclusion criteria followed were as follows:

1. Patient with cleft lip and palate
2. Patient with palatopharyngeal insufficiency
3. Patient with palatopharyngeal incompetency
4. Patient with history of maxillofacial surgery
5. Patient with muscle dystrophy or related disorder
6. Patients with any intraoral or extraoral pathology

(All these conditions may result in change in muscular anatomy and relation of soft palate with hard palate.)

After selection, from each subject written informed consent was obtained and then intraoral scans were performed.

**Figure 1:**
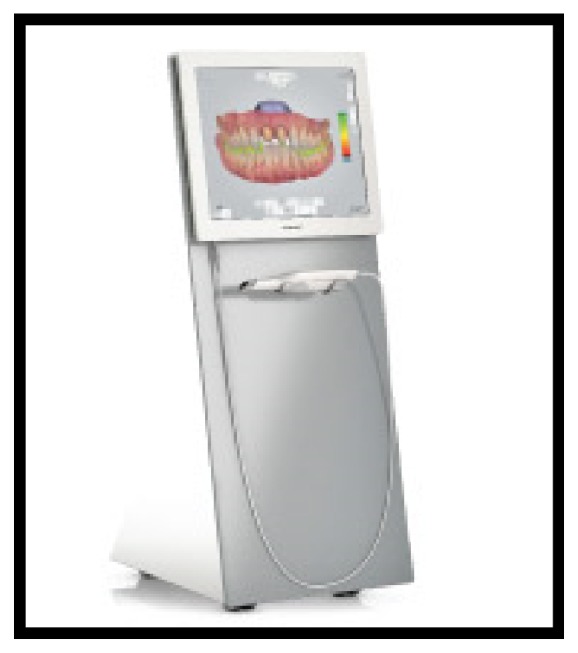
Intraoral scanner cart.

The classified instruments required for this study were digital intraoral scanner (3shape TRIOS–3 Shape A/S, Holmens Kanal 7, 4, 1060 Copenhagen K, Denmark) (Figure 1), 3D analysis software with computer (Minimagic 2, Materialize Technologielaan 15, 3001 Leuven, Belgium), and diagnostic set of instruments.

### 2.4. Methodology

The steps involved in the study were explained in detail to each selected subject. Patient were made to sit in dental chair in straight upright posture with neutral head position. Then instructions were given to the patient to open their mouth (minimum 22 mm, normal mouth opening). The intraoral scanner was used to make an extended distal impression of the maxillary arch. Care was taken to avoid any contact of the scanner with the tissues of the palatal area.

An extended scanning involving soft palate was recorded and saved in the computer as  .dcm file format (DICOM File Format, Digital Imaging and Communications in Medicine). The first recording was made to acclimatize the patient with the scanner as this was the new instrument compared to the other regularly used instruments in dentistry. This first recording makes the patient comfortable and more cooperative and chances of alteration in the angulation between soft and hard palate due to muscle movement (following stress of unknown instrument) were eliminated. The second recording was saved for assessment. Each recording took approximately 5–7 minutes. Even though both scans were the same and recorded same tissues most of the time in second scan the patients were familiar with the process and were relaxed, so the exact relation between soft and hard palate was recorded.

**Figure 2:**
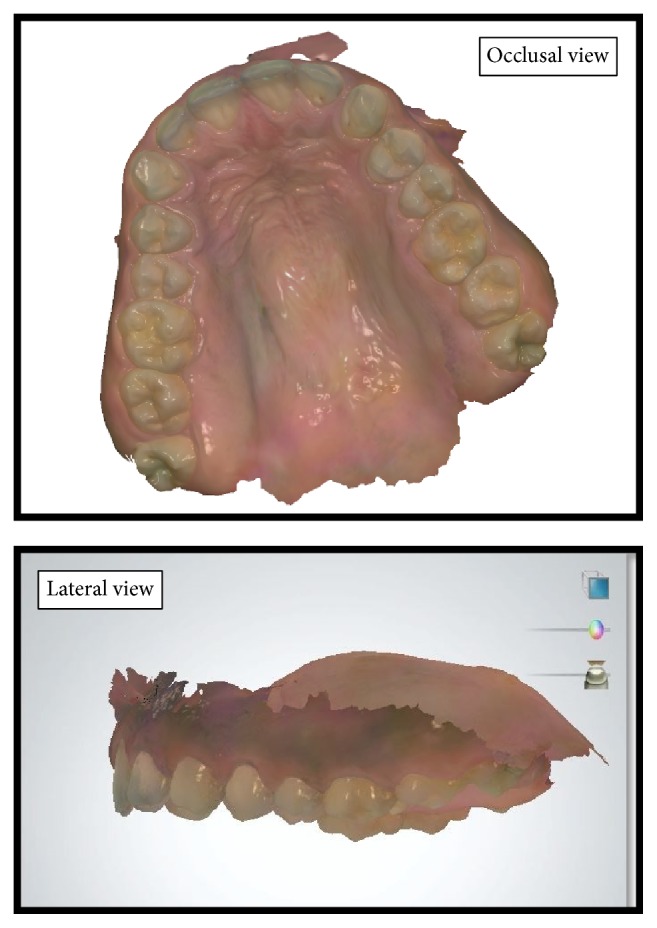
Screenshot of the scanned maxillae showing the extended scanning of soft palate. (occlusal view, lateral view).

All scans with the intraoral scanner were recorded by the same examiner same who was blinded to the purpose of the study. Each scanning was done in a predetermined order. Scanning started from the distal to the most posterior tooth in the first quadrant with an extended recording of the soft palate and continued to the anterior teeth. Next, the scanning was performed for the second quadrant, again beginning from distal to the most posterior tooth. Each tooth was scanned from its buccal and lingual sides by placing the camera at an angle of 45° to the tooth axis. Any voids in the scan were captured after the entire arch was scanned (Figure 2).

Images of each tooth showed neighboring parts of adjacent teeth. These served to overlap the pictures to create a virtual model of the whole arch from single images. All virtual models were exported through 3-shape communication to the laboratory computer; then the files were retrieved and exported as Standard Tessellation Language (STL; 3D Systems) format to new computer containing the 3D analysis software. With the help of this software the angulations between the hard palate and soft palate were measured and recorded under respective palatal throat form.

**Figure 3:**
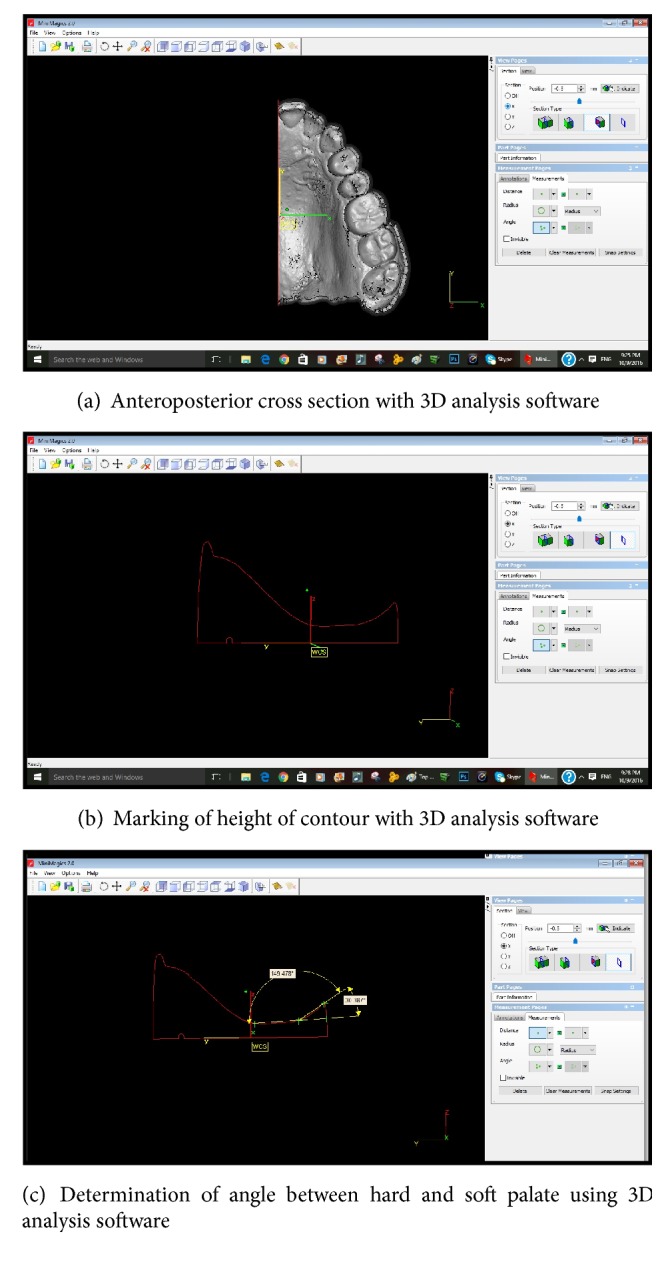


For measuring the exact angulation between the hard and soft palate the 3D picture so obtained after making the 3D digital impression was viewed from the right view panel and anteroposterior cross section through the midline was created (Figure 3(a)). Midline was determined by bisecting the line joining the canine tips of quadrants one and two by the line from the tip of the incisive papilla and extending it posteriorly up to the soft palate. Now in this the height of contour was marked on the hard palate and a tangent was drawn and extended posteriorly (Figure 3(b)). The soft palate was bisected with a line and extended to join the tangent of hard palate (Figure 3(c)). The angulation so formed was calculated and recorded according to classification (type I > 10, type II = 10–45, and type III > 45 degrees). The data so obtained was tabulated and statistically analyzed to determine the results.

## 3. Results

The data so obtained was entered in the computer and statistical procedures were performed using SPSS software version 16.0 (SPSS, Inc., Chicago, IL). The data were analyzed for descriptive statistics and inferential statistics using chi-square and Student's *t*-test.

**Figure 4:**
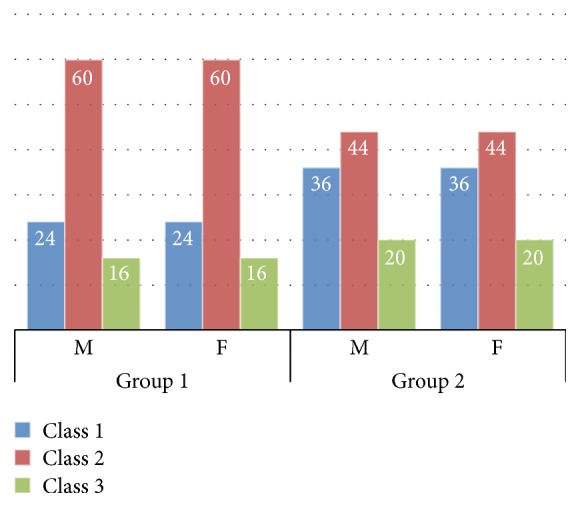
Percentage distribution of type of soft palate among groups.

The distribution of palatal throat form among groups was determined and is shown in Figure 4.

The results show that the prevalence of Class II type of soft palate (52%) was greatest followed by Class I (30%) and Class III (18%) (Table 2). Statistical analysis of the selected sample data showed significant difference (*p* value ~ 0) between Class I, II, and III types of soft palate.

In Table 3, it could be seen that in both the groups Class II type of soft palate has highest prevalence. The calculated chi-square value = 2.65 for 2 degrees of freedom (df) at 5% level of significance is found to be nonsignificant. There was no significant difference in the occurrence of soft palate types between age groups (*p* = 0.265).

On analyzing the gender distribution in individual class, it was observed that there is statistical difference between the male and female soft palate angulation in all classes at 5% level of significance (*p* = 0.000). Although the females had higher soft palate angulation in all classes with mean Class I, 8.20, Class II, 36.00, and Class III, 63.67, compared to the males with mean angulation Class I, 5.93, Class II, 28.15, and Class III, 55.00 (Table 4).

## 4. Discussion

Successful treatment planning in dentistry requires precise diagnostic information and an extensive diagnosis [13–15]. Without proper diagnosis treatment may go in vain. For years arbitrary methods were used for determination of soft palate type at chair side, in both dentulous and edentulous patients. Even though various studies recommended individual clinical examination, diagnostic cast, radiographic methods, and various combinations, for its correct determination still exact determination is beyond reach. Nevertheless radiographic methods provide little benefit over other methods but exposure to radiation, overlapping of images, and soft tissue radiolucency are major concern. In dental model analysis, plaster models have been the standard for years. However, plaster models are subjected to variations in dimensions and reproducibility and accuracy at soft tissue region. Especially in case of palatal throat form determination the use is highly questionable.

In this study, the intraoral digital impression system was used owing to its reproducibility in virtual image form, of hard and soft tissues without physical contact, and accuracy of soft tissue region its precision compared with conventional techniques and extraoral digitization of stone casts. Moreover the use of intraoral scanner for scanning directly in the patient's mouth made dental impressions obsolete [31, 32]. This can be advantageous for patients with a gag reflex or with cleft lip and palate, who are at risk of aspiration and respiratory distress during taking of the dental impressions [3, 33, 34]. Thus by pioneering the use of intraoral scanner in this study, it had not only assisted in meticulously fulfilling the aim of the research but also opened new channels in diagnosis and treatment planning.

As described above, soft palate plays a vital role not only in normal physiological functions but also during fabrication of prosthesis in various situations like complete dentures, obturators, palatal lift prosthesis, sleep apnea, and so on [1–6, 12–14]. Correct and exact identification of type of soft palate is of vital significance in diagnosis treatment planning and prognosis. This study uses digital impression by which exact angulation between hard and soft palate was determined with the help of 3D analysis software. At community level the results of the study will be helpful in diagnosis and treatment of above said conditions, for example, speech disorder, sleep apnea, and cleft palate. The treating dentist will know the prevalence of the type of soft palate in the targeted population so accordingly the diagnosis and future treatment and fabrication of prosthesis can be managed. Studies reveal that there is an increase in length, thickness, and sagittal area of soft palate with age [1, 4] in both the genders but increase in soft palate area was significantly more in males [7, 8].

In the present study, the groups were made on the basis of age. Age ranges were taken into consideration to facilitate the collection of samples and to avoid any particular result by chance in particular age.

It has been agreed that the posterior palatal seal area (PPSA) is one of the dominant factors which determines the success of complete denture. Type of soft palate plays an important role in establishing the posterior palatal seal in complete denture. In order to correctly locate PPSA, careful observation and palpation of the tissues are necessary, as their locations vary with the contours of the soft palate. Thus, a combination of the palpatory and visual methods has been used in clinical situations to determine the type of soft palate [12–14, 19] which mostly depends on the experience of the clinician. But this method incorporates the operator's bias as well as patient bias. The method described in the present study is completely bias-free and helps in determining the exact angulation and type of soft palate, by using 3-dimensional pictures of the hard and soft palate generated by using intraoral scanner. With the correct knowledge of the type of soft palate at the diagnosis stage the clinician would be able to record the PPSA properly at the time of final impression of complete denture fabrication. This is in contrast with the previous studies which were of little use as the lateral cephalogram with overlapping images was used and to calculate angulations the angle was created between the base of the hard palate and the soft palate, rather than between the surfaces of the soft and hard palates [19, 35–39].

Recent advances in radiodiagnostic technique like CBCT (cone-beam computed tomography) have allowed its commercial production and practical application in today's patient care and dental education environment [23]. It has added advantages over other radiodiagnostic techniques like giving three-dimensional view of an object and correct identification of landmarks as compared to lateral cephalogram which gives two-dimensional view of an object. The linear measurements that are made from CBCT images are not significantly different from actual direct measurements of anatomic structures in the dentomaxillofacial area but, owing to their cost and availability at chairside for diagnosis, are measure concern [40, 41]. In the present study the simple intraoral scanner overweighed CBCT and proved helpful. CBCT can give the clinician a 3D representation of the teeth, but its accuracy and reliability for dental measurements have not been fully assessed. Past studies have analyzed CBCT accuracy of craniofacial landmarks and determined that measurements were statistically significantly different from measurements taken with a digital caliper but still clinically acceptable (90% of mean differences, 2.00 mm) [42, 43]. Baumgaertel et al. [44] studied reliability and accuracy of cone-beam computed tomography dental measurements and concluded that dental measurements from CBCT volumes can be used for quantitative analysis. They specified with CBCT images a small systemic error, which became statistically significant only when combining several measurements. According to our knowledge no one till now compared the soft palate angulation with CBCT.

The use of 3-dimensional (3D) analysis with “3D compare” software commands (3D compare analysis) has become a norm within reverse engineering and has been adopted within the field of dentistry [45–50]. The use of mathematical software 3D analysis software proved beneficial in the present study as it helped in calculation of the angulation in a highly precise manner without any bias, thus overcoming the manual measurement of angulations. The use of computer technology in our study excelled the methodology to its highest point where the soft tissues were manipulated without any direct contact of the tissues to be recorded (especially at the palatal area) thus resulting in zero deformation, so ascertaining the preciseness of the study and its use in diagnosis and treatment planning.

The results of the study showed that Class II type of soft palate is the most common and high in prevalence in the targeted population in Aseer Province, with 60% in group 1 and 44% in group 2; this finding is in contrast with the previous available conducted studies where Class I type of soft palate was most common [12–14, 16]. Such variation may be due to genetic differences.

Statistical significant difference was found between the classes (Table 2) which is in association with previous description by House [16], Winkler [13], and Boucher et al. [14] as there was pronounced difference in angulation between different classes of soft palate. This also emphasized the benefit of using the intraoral scanner for the study as precise determination of angulation was possible.

There was significant difference between types of soft palate between males and females in each class of soft palate (Table 3) and with age no significant difference was noted in angulation with hard plate (Table 4) determining that age had no effect on the angulation between hard and soft palate. The female had higher angulation between soft and hard palate as compared with the males in all the classes and in both groups this may also be due to genetic development of the studied population.

### 4.1. Limitations

However, limitation of this study includes restricted mouth opening and size of the intraoral scanner. If the intraoral scanner touched any part of the palate during recording there may be movement in the soft palate and thus angulation varies which may affect the final outcome. According to the scanning protocol, a fixed number of pictures are acquired of every section. In the present study extended scanning protocol was accepted, resulting in a lengthier scanning time, which was essential to obtaining comprehensive information. With the intraoral scanner we used, most patients seemed to be uncomfortable while the buccal surfaces of the maxillary second and third molars were scanned. The discomfort was related to the dimensions of the scanning tip and its interference with the patient's coronoid process. As scanning technology continues to evolve, the design of a thinner scanning tip may improve comfort and increase patient acceptance of the scanning procedure. Any new technology will gain wider acceptance only if it provides significant advantages over established methods. So, further studies should be carried out with still smaller size of intraoral scanner and larger sample size to identify the exact scenario in the population.

## 5. Conclusion

The present study highlighted the extended use of intraoral scanner and determined the type of soft palate present in the targeted population in Aseer Province. Among the various types of soft palate, type II soft palate was more common in area of concern. The study also implicated that higher soft palate angulation was present among females compared to males in both the groups. The age changes had no effect in the type of soft palate in the region.

## Figures and Tables

**Table 1:** Division of samples in group.

Group 1		Group 2	
50 samples		50 samples	
20–40 years		41–60 years	
25 male	25 female	25 male	25 female

**Table 2:** Distribution of soft palate types.

	Total (*N*)	Chi-square value	df	*p* value
Class I	30	17.840	2	0.000^*∗*^
Class II	52			
Class III	18			

Total	100			

^*∗*^Significant at 5% level of significance.

**Table 3:** Distribution of soft palate types by age group.

	Class I (*N*)	Class II (*N*)	Class III (*N*)	Total	Chi-square value	df	*p* value
Age of 20–40 years Group 1	12	30	8	50	2.65	2	0.265^*∗*^
Age of 40–60 years Group 2	18	22	10	50			

Total	30	52	18	100			

^*∗*^Significant at 5% level of significance.

**Table 4:** Comparison between male and female in different classes of soft palate.

	Gender	*N*	Mean	Std. deviation	*T*-value	df	*p* value
Class 1	Male	15	5.93	1.486	−4.194	28	0.000^*∗*^
	Female	15	8.20	1.474			

Class 2	Male	26	28.15	7.176	−4.341	50	0.000^*∗*^
	Female	26	36.00	5.783			

Class 3	Male	9	55.00	6.164	−3.159	16	0.006^*∗*^
	Female	9	63.67	5.454			

^*∗*^Significant at 5% level of significance.
